# Down‐regulation of miR‐10a‐5p in synoviocytes contributes to TBX5‐controlled joint inflammation

**DOI:** 10.1111/jcmm.13312

**Published:** 2017-08-07

**Authors:** Nazim Hussain, Wenhua Zhu, Congshan Jiang, Jing Xu, Xiaoying Wu, Manman Geng, Safdar Hussain, Yongsong Cai, Ke Xu, Peng Xu, Yan Han, Jian Sun, Liesu Meng, Shemin Lu

**Affiliations:** ^1^ Department of Biochemistry and Molecular Biology School of Basic Medical Sciences Xi'an Jiaotong University Health Science Center Xi'an Shaanxi China; ^2^ Key Laboratory of Environment and Genes Related to Diseases (Xi'an Jiaotong University) Ministry of Education Xi'an Shaanxi China; ^3^ Department of Joint Surgery Xi'an Hong Hui Hospital Xi'an Jiaotong University Health Science Center Xi'an China

**Keywords:** arthritis, microRNA‐10a‐5p, TBX5, synoviocytes

## Abstract

MicroRNAs are considered to play critical roles in the pathogenesis of human inflammatory arthritis, including rheumatoid arthritis (RA). The purpose of this study was to determine the relationship between miR‐10a‐5p and TBX5 in synoviocytes and evaluate their contribution to joint inflammation. The expression of miR‐10a‐5p and TBX5 in the synovium of RA and human synovial sarcoma cell line SW982 stimulated by IL‐1β was determined by RT‐qPCR and Western blotting. The direct interaction between miR‐10a‐5p and TBX5 3′UTR was determined by dual‐luciferase reporter assay in HeLa cells. Mimics and inhibitors of miR‐10a‐5p were transfected into SW982 cells. TBX5 was overexpressed by plasmid transfection or knocked down by RNAi. Proinflammatory cytokines and TLR3 and MMP13 expressions were determined by RT‐qPCR and Western blotting. Down‐regulated expression of miR‐10a‐5p and up‐regulation of TBX5 in human patients with RA were found compared to patients with OA. IL‐1β could reduce miR‐10a‐5p and increase TBX5 expression in SW982 cells *in vitro*. The direct target relationship between miR‐10a‐5p and 3′UTR of TBX5 was confirmed by luciferase reporter assay. Alterations of miR‐10‐5p after transfection with its mimic and inhibitor caused the related depression and re‐expression of TBX5 and inflammatory factors in SW982 cells. Overexpression of TBX5 after pCMV3‐TBX5 plasmid transfection significantly promoted the production of TLR3, MMP13 and various inflammatory cytokines, while this effect was rescued after knocking down of TBX5 with its specific siRNA. We conclude that miR‐10a‐5p in a relation with TBX5 regulates joint inflammation in arthritis, which would serve as a diagnostic and therapeutic target for RA treatment.

## Introduction

Rheumatoid arthritis (RA) has been commonly recognized as a chronic inflammatory autoimmune disease affecting about 0.5–1% of the human population throughout the world 1, 2. RA, a systemic disease characterizes by joint inflammation and demolition in which fibroblast‐like synoviocytes (FLS), residing in synovial joints, plays critical roles in the induction of inflammation, the progressive destruction of cartilage and bone and formation of pannus 3, 4, 5. After the onset of RA, FLS show an abnormal behaviour as their apoptotic, proliferative and invasion properties changed. They increase in numbers and along with other immune cells produce an imbalance in immune homeostasis, which causes to establish the inflammatory milieu in synovium, resulting in more damage to bone and cartilage 6, 7, 8. The activated FLS have been considered as the primary source for the secretion of proinflammatory cytokines and chemokines, the recruitment of inflammatory cells and the production of matrix metalloproteinases (MMPs) 9, 10, 11, 12.

MicroRNAs (miRNAs) are evolutionarily conserved, a class of small, 20–22 nucleotide long, noncoding endogenous RNA molecules that bind to the 3′UTR of their target genes, leading to either translational delay or mRNA degradation 13, 14, 15. miRNAs regulated gene expression is a novel and important mechanism governing the expression of a significantly large portion of the genome. It has been proposed that approximately 30% of the human protein‐coding genes are controlled by miRNAs 16, 17. miRNAs are involved in several biological processes such as proliferation, differentiation, apoptosis, development, angiogenesis and immune response *via* regulating their target genes 18. The abnormal expression of miRNAs participates in the pathogenesis of several human diseases including RA 19, 20, 21, 22, 23, 24.

At present, some miRNAs have been found to be dysregulated in patients with RA, for instance, miR‐146a, miR‐155, miR‐203 and miR‐346 were up‐regulated in synovial fluids, fibroblast and peripheral blood mononuclear cells of patients with RA but miR‐124a was found to be down‐regulated in RA 25, 26, 27. MiR‐23b as an anti‐inflammatory miRNA was also down‐regulated in RA compared to OA 28. MiR‐146a was found to cause the extended production of TNF‐α in patients with RA, and later evidence showed that the expression of miR‐146a could be up‐regulated by TNF‐α in CD4^+^ T cells of patients with RA 29, 30. MiR‐19b could cause the production of proinflammatory cytokines and inflammation through regulating NF‐κB 31. Up‐regulated expression of miR‐223 has been described in CD4^+^ naive T lymphocytes of patients with RA as compared to healthy ones. There is an evidence of intensive expression of miR‐223 in RA synovium due to its increased number of positive cells in RA synovium and this overexpression caused the suppression of osteoclastogenesis *in vitro*. So miR‐223 shows its role in the treatment of bone destruction in patients with RA 32, 33. Decreased expression of miR‐124a increased the expression of CDK‐2 which promoted the secretion of MCP‐1 and an elevation in RA synovial cell proliferation and inflammation 34. MiR‐573 has been found to suppress the activation of MAPK which is considered as one of the potential targets for the treatment of RA, so it plays like a negative regulator in RA 35. With the help of high‐throughput sequencing technologies, more and more miRNA dysregulation would be revealed. However, the precise molecular mechanism underlying how the dysregulated miRNAs influence RA succession remains to be elucidated.

In our previous work, we tried to investigate the mechanism of joint inflammation caused by arthritogenic T cells using pristane‐induced arthritis (PIA) which is a T cell‐dependent rat model. We co‐cultured rat FLS with splenic T cells and found that pristane‐primed rat T cells enhance TLR3 expression of FLS *via* TNF‐α initiated p38 MAPK and NF‐κB pathways 36. Subsequent study using miRNA microarray showed that the miRNA profile in FLS was changed due to T cell stimulation, and particularly miRNA‐10a‐5p was found to be down‐regulated. MiR‐10a‐5p has been found to be dysregulated in immune cells and involved in autoimmune diseases *via* restricting inflammation 37, 38, 39, 40, 41. However, the function of miR‐10a‐5p in the activation of joint inflammation and particularly in the interaction between T cells and FLS remains poorly understood. The relationship between miRNAs and its target genes is considered important for understanding the regulatory mechanism of miRNAs in the development of autoimmune diseases like RA. TBX5 was selected as one of the potential target genes of miR‐10a‐5p, which is an essential transcription factor with multiple roles even in inflammation. Thus, the aim of this study was to explore the role of miR‐10a‐5p through its target gene TBX5 in the pathogenesis of RA.

## Materials and methods

### Ethics statement

All experimental procedures regarding specimen collection from human participants were approved by the Human Research Protective Committee (Xi'an Jiaotong University Health Science Center). In addition to collecting all clinical and non‐clinical information required in this project, a written informed consent was also taken from all patients.

### Human samples

Human synovium samples were collected from patients with RA (six men and eight women, 38–70 years old) undergoing joint replacement at Xi'an Hong Hui Hospital. Fourteen osteoarthritic patients' samples (eight men and six women, 35–65 years old) were used as controls. Synovial tissue samples were collected, and mRNA and protein were extracted for gene expression assay.

### Cells

Human synovial sarcoma cell line SW982 and HeLa cells were cultured in DMEM supplemented with 10% FBS. All cells were incubated at 37°C in humid condition provided with 5% CO_2_.

### Cytokine stimulation

SW982 cells (2 × 10^5^ cells/ml) were seeded into 6‐well plates until a confluence of cells was reached between 70% and 80%. Cells were then stimulated with IL‐1β or TNF‐α (10 ng/ml). The cells were then harvested after 24 hrs for RNA extraction and at 48 hrs for protein extraction.

### Transfection with miR‐10a‐5p mimic and inhibitor

FLS were cultured in 12‐well or 6‐well plates 24 hrs earlier to transfection. miRNA control mimic (5′ UUG UAC UAC ACA AAA GUA CUG 3′), miR‐10a‐5p mimic (5′ UAC CCU GUA GAU CCG AAU UUG UG 3′), miRNA control inhibitor (5′ CAG UAC UUU UGU AGU ACA A 3′) and miR‐10a‐5p inhibitor (5′ CAC AAA UUC GGA UCU ACA GGG UA 3′) (GenePharma, China) were transfected at a final concentration of 50 nM with Lipofectamine 2000 (Invitrogen, USA). After 24 and 48 hrs of transfection, RNA and protein were extracted respectively.

### Luciferase reporter assay

TBX5 3′UTR segment was cloned by PCR (Forward primer: GCG GAG CTC GAA ATG AAA CCC AGC ATA; reverse primer: GCG AAG CTT AGC CTC ACA TCT TAC CCT), and then inserted into downstream of the luciferase gene between Sac*I* and Hind*III* sites within the pMIR‐Report™ luciferase vector (Ambion, Austin, USA). The pRL‐TK vector (Promega, Fitchburg, USA) was used as a control. All plasmid vectors were extracted with the EZNA™ Endo‐free Plasmid Maxi Kit (Omega BioTek, Norcross, USA). The constructed vectors were then sent to the company (GenScript Company, Nanjing, China) for the verification of sequence integrity.

HeLa cells were cultured in a 48‐well plate containing DMEM high‐glucose medium (HyClone, USA) supplemented with 10% FBS (HyClone) 24 hrs before transfection. Then cells (0.5 × 10^5^ cells per well) were transfected with firefly pMIR‐Report™ luciferase (Ambion) and Renilla pRL‐TK (Promega) vectors (90 ng:10 ng per well) and at the same time with 50 nM miRNA control mimic or 50 nM miR‐10a‐5p mimic (GenePharma, China). The Renilla luciferase reporter was used for normalization. After 24 hrs of transfection, the cells were lysed, and luciferase activity was detected using Dual‐Luciferase^®^ Reporter 1000 Assay System (Promega) by a plate‐reading luminometer (Tecan).

### Intervention of TBX5

For the knock‐down expression of TBX5 in SW982 cells, si‐TBX5 (F: 5′ GGG CAC GGA AAU GAU CAU ATT 3′; R: 5′ UAU GAU CAU UUC CGU GCC CTT 3′) and si‐NC (F: 5′ UUC UCC GAA CGU GUC ACG UTT 3′; R: 5′ ACG UGA CAC GUU CGG AGA ATT 3′) were purchased from Shanghai GenePharma. To overexpress the TBX5 expression in SW982 cells, the overexpressing TBX5 plasmid pCMV3‐TBX5‐GFPSpark and control vector (pCMV3‐C‐GFPSpark‐CV) were purchased from Sino Biological Inc. These plasmids were then transfected to DH5α, and constructs were prepared using EZNA™ Endo‐free Plasmid Maxi Kit (Omega BioTek, Norcross, USA).

### RNA quantification

SW982 cells were collected after various treatments. MRNA expression of different genes and miRNA level was determined by RT‐qPCR. Total RNA was extracted with TRIzol^®^ Reagent (Invitrogen), quantified using NanoDrop. A total RNA of 0.5 μg was utilized in a miRNA‐specific stem‐loop reverse transcription (RT) reaction for miRNAs, and 2 μg for the RT reaction using oligo d(T) primer. Then cDNA was synthesized by RevertAid™ First Strand cDNA Synthesis Kit (Fermentas). Real‐time quantitative PCR (qPCR) was performed by iQ5 (Bio‐Rad) with SYBR^®^ Premix Ex Taq™ *II* (TaKaRa) for quantification. The expression of genes and miRNAs was normalized by β‐actin and U6 snRNA, respectively. All data were analysed using 2^−ΔΔCt^ (relative quantification) method. The information about genes, primer sequences, and annealing temperatures has been depicted in Table 1.

**Table 1:** List of primers for RT‐qPCR

Genes	Sequences	Ta (°C)
*miRNA‐10a‐5p* (RT)	GTCGTATCCAGTGCAGGGTCCGAGGTATTCGCACTGGATACGACCACAAA	–
*miRNA‐10a‐5p*	F: CGCTACCCTGTAGATCCGAA	60
	R: GTGCAGGGTCCGAGGT	
*U6*	F: CTCGCTTCGGCAGCACA	60
	R: AACGCTTCACGAATTTGCGT	
*TBX5*	F: GAGATAGTCGCTATCGCCTGG	60
	R: AGGTTCTGCTCTCCAACTATCC	
*TLR3*	F: GCTAGCAGTCATCCAACAGAATC	60
	R: AGTCAACTTCAGGTGGCTGC	
*TNF‐*α	F: CCAGGCAGTCAGATCATCTTCTC	59
	R: GGAGCTGCCCCTCAGCTT	
*IL‐1*β	F: ACAGATGAAGTGCTCCTTCCA	59
	R: GTCGGAGATTCGTAGCTGGAT	
*IL‐8*	F: TCTGCAGCTCTGTGTGAAGG	60
	R: AAATTTGGGGTGGAAAGGTT	
*IL‐6*	F: CAATCTGGATTCAATGAGGAGAC	60
	R: CTCTGGCTTGTTCCTCACTACTC	
*MMP13*	F: TGCTGCATTCTCCTTCAGGA	59
	R: ATGCATCCAGGGGTCCTGGC	
*Βeta‐ACTIN*	F: CATCTCTTGCTCGAAGTCCA	59
	R: ATCATGTTTGAGACCTTCAACA	

### Western blotting

For Western blotting analysis, synovial tissue and SW982 cells were lysed with RIPA lysis buffer, and protein was extracted. Total protein was then quantified using BCA kit (Thermo, USA). All protein samples with equal amounts about 30 μg were loaded on a 10% SDS‐denatured polyacrylamide gel (SDS‐PAGE) and then transferred to polyvinylidene difluoride membranes (Amersham, Buckinghamshire, UK). After 2 hrs of blocking with 5% fat‐free milk the membranes were then subsequently incubated with polyclonal anti‐TBX5 antibody (1:500, Abcam, USA), polyclonal anti‐TLR3 antibody (1:200, Proteintech, Chicago, USA), polyclonal anti‐MMP13 antibody (1:250, Abcam, USA) or GAPDH (1:10,000, Proteintech, Chicago, USA) overnight. The membranes were then washed with 1× TBST and incubated with a horseradish peroxidase‐conjugated (HRP‐conjugated) secondary antibody for 2 hrs. Protein expression was evaluated by SuperSignal^®^ West Pico kit (Thermo Scientific, Carlsbad, CA, USA).

### Statistical analyses

The experimental data were presented as mean ± standard error of the mean (S.E.M.), and the statistically significant difference between the experimental and control groups was then determined using Student's *t*‐test. *P *< 0.05 was considered to be statistically significant.

## Results

### MiR‐10a‐5p expression is down‐regulated, and TBX5 expression is up‐regulated in the synovium of patients with RA

**Figure 1:**
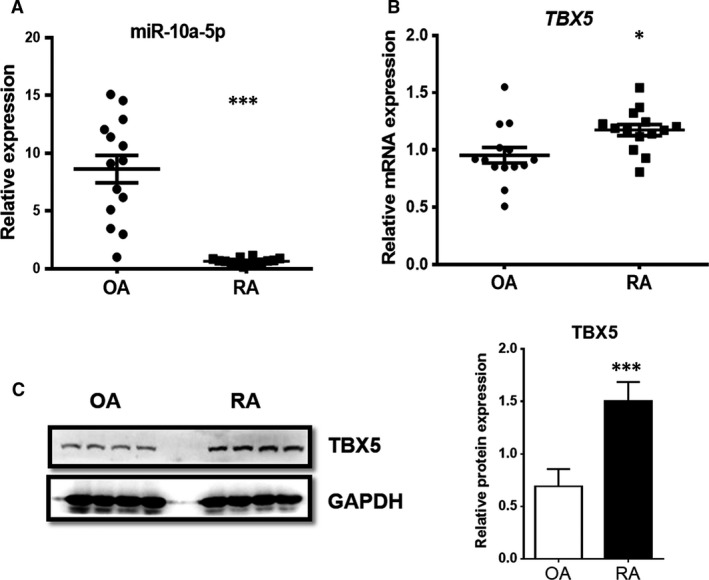
Expression levels of miR‐10a‐5p and TBX5 in the synovium of human patients with RA and OA. Relative mRNA expression levels of mature miR‐10a‐5p (**A**) and TBX5 (**B**) in the synovium of human patients with RA and OA (*n *=* *14) were evaluated by RT‐qPCR. Housekeeping genes U6 snRNA and β‐actin were used for the normalization of miRNA and mRNA, respectively. Relative protein expression levels of TBX5 (**C**) was detected by Western blotting analysis and normalized by GAPDH. Data represent the means ± S.E.M. of three independent experiments. Levels of significance were calculated using Student's *t*‐test (*P < 0.05, ***P < 0.001).

We had co‐cultured rat FLS with splenic T cells isolated from PIA rats or control rats respectively, and then miRNA microarray analysis was performed to determine the epigenetic changes in FLS caused by T cells. Interestingly, our results showed 57.5% more down‐regulation in the expression level of miR‐10a‐5p in FLS co‐cultured with pristane‐primed T cells *versus* control T cell group. Therefore, in the present study, we tried to confirm the regulation of miR‐10a‐5p in patients with RA as well as in human synoviocyte cell line SW982, and further investigate its functions. The expression levels of miR‐10a‐5p and TBX5 in the synovium of patients with RA and patients with OA (controls) were detected using RT‐qPCR and Western blotting analysis. It was found that miR‐10a‐5p expression in patients with RA was significantly down‐regulated as compared to patients with OA (Fig. 1A). miRNA database (TargetScan) was used, and TBX5 was selected as one of the possible candidate target genes of miR‐10a‐5p, and noticeably elevated expression of TBX5 was detected in synovium of patients with RA as compared with patients with OA (Fig. 1B and C). These data suggested that a decline in miR‐10a‐5p expression and an elevation in TBX5 expression might be involved in the progression of RA.

### IL‐1β leads to regulation of miR‐10a‐5p and TBX5 in FLS

**Figure 2:**
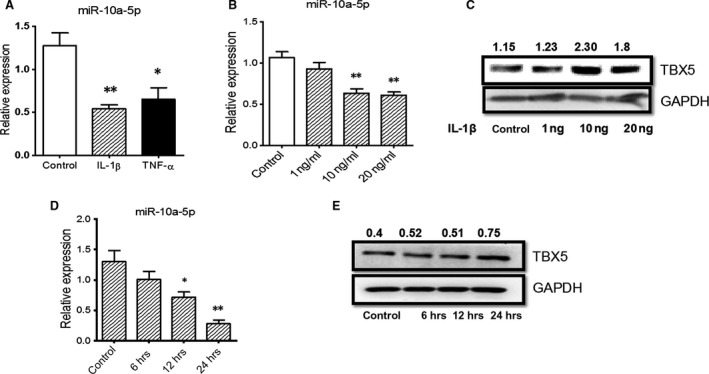
IL‐1β regulated the expression of miR‐10a‐5p and TBX5 in SW982 cells. SW982 cells were stimulated with IL‐1β and TNF‐α for 24 hrs, and relative expression of miR‐10a‐5p (**A**) was detected by RT‐qPCR. The cells were then stimulated with different doses of IL‐1β for 24 hrs (**B** and **C**) or with 10 ng/ml IL‐1β for different time‐points (**D** and **E**), respectively. miRNA expression was detected by RT‐qPCR, whereas TBX5 expression was determined by Western blotting. The expression levels of miR‐10a‐5p and TBX5 were normalized by U6 snRNA and GAPDH, respectively. The data represent the means ± S.E.M. of three independent experiments. Levels of significance were calculated using Student's *t*‐test (*P < 0.05, **P < 0.01).

Regulation of miR‐10a‐5p in rat FLS should result from T cell‐derived inflammatory stimulation in our previous model. Evidence shows that the expression of miRNAs can be regulated by inflammatory signals 42, 43. MiR‐10a‐5p showed significant down‐regulation when SW982 cells, a human FLS cell line, were stimulated with IL‐1β and TNF‐α, the classical proinflammatory cytokines, and IL‐1β compared to TNF‐α was found to be more efficient for the down‐regulation of miR‐10a‐5p in our cell model (Fig. 2A). Then SW982 cells were stimulated with different concentrations of IL‐1β, and results showed the dose‐dependent down‐regulation of miR‐10a‐5p and up‐regulation of TBX5, respectively (Fig. 2B and C). MiR‐10a‐5p down‐regulation and TBX5 up‐regulation were also found under time‐dependent stimulation of IL‐1β in SW982 cells (Fig. 2D and E). On the other hand, although TNF‐α could also reduce miR‐10a‐5p expression, we found that it could not up‐regulate the expression of TBX5 smoothly, or even reduce the TBX5 expression after 12‐h stimulation (Data not shown). These data suggested that IL‐1β was the inflammatory mediator leading to miR‐10a‐5p down‐regulation.

### MiR‐10a‐5p directly targets TBX5

**Figure 3:**
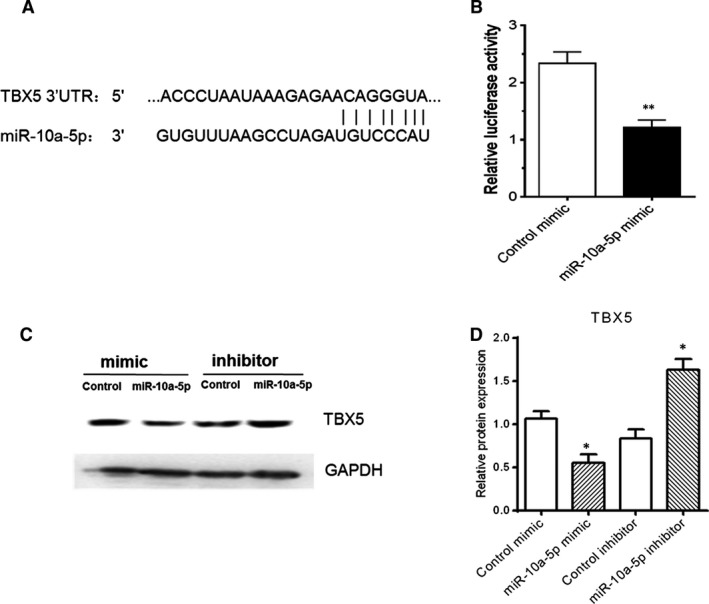
TBX5 was a direct target of miR‐10a‐5p. (**A**) The schematic graph showed pairing relationship between miR‐10a‐5p and TBX5 mRNA 3′UTR in human. Target relationship between TBX5 mRNA 3′UTR and miR‐10a‐5p (**B**) was detected by dual‐luciferase reporter assay. For the luciferase reporter assay, HeLa cells were cotransfected with the luciferase reporter vectors (pMIR‐REPORT™) containing TBX5 3′UTR along with control mimic or miR‐10a‐5p mimic. After 48 hrs of transfection, relative luciferase activity was measured. HeLa cells were transfected with 50 nM miR‐10a‐5p mimics or 50 nM miR‐10a‐5p inhibitors for 48 hrs, and TBX5 expression (**C** and **D**) was measured by Western blotting analysis. The data represent the means ± S.E.M. of three independent experiments. Levels of significance were calculated using Student's *t*‐test (*P < 0.05, **P < 0.01).

miRNAs regulate various cellular activities and biological functions by regulating the expression of their target genes. Bioinformatics tools such as TargetScan Release 5.2 and miRBase targets were used to investigate the relationship between miR‐10a‐5p and TBX5. We identified TBX5 as a potential target gene of miR‐10a‐5p. To verify the binding site, the 3′UTR of TBX5 comprising the seed sequence of miR‐10a‐5p was cloned and used in a firefly luciferase reporter assay (Fig. 3A). To determine the relative luciferase activity, HeLa cells were cotransfected with reporter plasmids containing TBX5‐UTR along with miR‐10a‐5p mimic or control mimic. The obtained data showed that the relative luciferase activity was significantly inhibited by miR‐10a‐5p mimic (Fig. 3B).

The stability and the translation of target gene can be regulated by miRNAs at the post‐transcriptional level 44. Protein expression level was detected by Western blotting analysis in HeLa cells after transfection with mimics and inhibitors of miR‐10a‐5p along with their controls. The obtained results showed that TBX5 protein expression level was decreased in miR‐10a‐5p mimic‐transfected cells while elevated in miR‐10a‐5p inhibitor‐transfected cells (Fig. 3C and D).

### Intervention of miR‐10a‐5p could regulate TBX5 and synoviocyte inflammation

**Figure 4:**
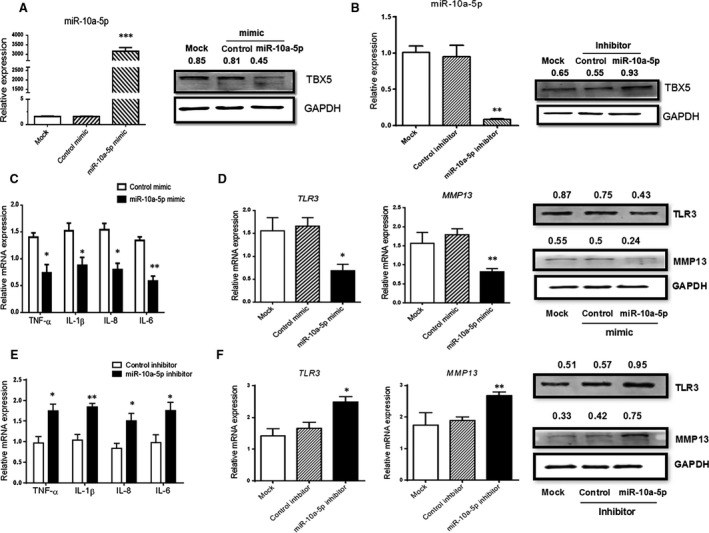
MiR‐10a‐5p influenced TBX5 expression negatively, regulating local inflammation. SW982 cells were transfected with miR‐10a‐5p mimics or miR‐10a‐5p inhibitors respectively, stimulating with 10 ng/ml IL‐1β. The expression of miR‐10a‐5p and TBX5 (**A** and **B**), expression of proinflammatory cytokines (**C** and **E**) and expression of TLR3 and MMP13 (**D** and **F**) were determined by RT‐qPCR and Western blotting after 24 hrs or 48 hrs of transfection. The data represent the means ± S.E.M. of three independent experiments. Levels of significance were calculated using Student's *t*‐test (*P < 0.05, **P < 0.01, ***P < 0.001).

We hypothesized the involvement of miR‐10a‐5p in the induction of proinflammatory cytokines, TLR3 and MMP13 as these mediators have their role in inflammation. SW982 cells were transfected with mimic or inhibitor of miR‐10a‐5p, and the efficiency of the regulation of miRNA and its target gene TBX5 was re‐confirmed by RT‐qPCR and Western blotting (Fig. 4A and B).

The elevated expression level of miR‐10a‐5p resulted in down‐regulation of proinflammatory cytokines (IL‐6, IL‐8, TNF‐α and IL‐1β), TLR3 and MMP13 (Fig. 4C and D). The decreased expression of miR‐10a‐5p caused the significant up‐regulation of inflammatory cytokines (IL‐6, IL‐8, IL‐1β and TNF‐α), TLR3 and MMP13 (Fig. 4E and F). The obtained results showed that the intervention of miR‐10a‐5p could induce the inflammation process through inflammatory cytokines and might contribute to the pathogenesis of RA.

### TBX5 regulation is involved in production of inflammatory cytokines in SW982

**Figure 5:**
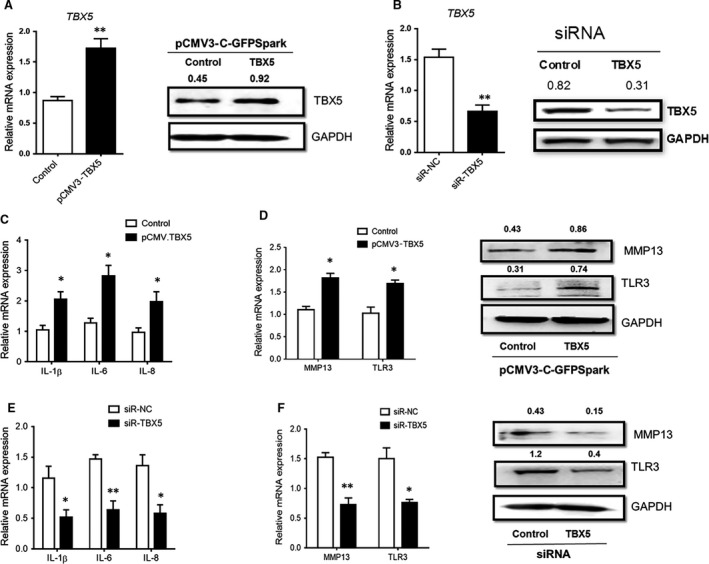
TBX5 mediated the downstream inflammation in IL‐1β‐stimulated SW982 cells. SW982 cells were transfected with overexpressing plasmid pCMV3‐TBX5‐GFPSpark or siRNA‐TBX5 to up‐regulate or down‐regulate its expression levels, respectively, and both mRNA and protein expression levels of TBX5 were confirmed by RT‐qPCR and Western blotting (**A** and **B**). Then IL‐1β was used to stimulate SW982 cells which were already transfected with overexpressing plasmids or siRNAs, and expression levels of cytokines (**C** and **E**), and TLR3 and MMP13 (**D** and **F**) were detected by RT‐qPCR and Western blotting. The data represent the means ± S.E.M. of three independent experiments. Levels of significance were calculated using Student's *t*‐test (*P < 0.05, **P < 0.01).

The above‐mentioned results showed that miR‐10a‐5p directly targeted TBX5 and induced inflammatory changes in SW982 cells. Then, we postulated that dysregulation of TBX5 might be involved in this inflammatory process. To confirm this hypothesis, SW982 cells were either transfected with overexpressing plasmids of the TBX5 pCMV3‐TBX5‐GFPSpark expression vector to enhance its expression or transfected with siRNA‐TBX5 to knock‐down its expression. The regulation efficiency was confirmed by RT‐qPCR and Western blotting (Fig. 5A and B). Overexpression of TBX5 promoted the expression levels of TLR3, MMP13 and inflammatory cytokines (Fig. 5C and D). Knocking down of TBX5 in SW982 cells resulted in the down‐regulation of TLR3, MMP13 and proinflammatory cytokines (Fig. 5E and F). Taken together, the obtained results illustrated that TBX5 participated in the production of inflammatory factors during joint inflammation, which we had proved to be induced by down‐regulation of miR‐10a‐5p.

## Discussion

RA is a chronic inflammatory autoimmune disease in which synovial fibroblasts are considered as important effector cells for joint destruction 3, 45. miRNAs are recently found noncoding small RNA molecules 46, which can regulate mRNA expression of the specific target gene at post‐transcriptional level and can control cell activation and differentiation processes of a variety of immune cells 47. It has been reported that the proliferation and invasion of RA FLS are affected by miRNAs 48. Pristane‐induced arthritis is a T cell‐dependent rat model 49, and infiltration of joint‐specific T cells into joints is involved in mediating local inflammation. In our previous study, we co‐cultured pristane‐primed T cells with normal FLS and found that T cell‐derived soluble cytokines contributed to TLR3 regulation and FLS activation. However, more work was needed to reveal the whole picture of the mechanism between T cells and FLS. Then, a miRNA microarray analysis had been performed in FLS co‐cultured with T cells, and miRNA‐10a‐5p showed significant down‐regulation. Several research articles have been published which support the evidence of deregulation of miR‐10a in human cancers, like gastric cancer, breast cancer and lung carcinoma, and in cervical tumour tissues with lymph node metastasis 50, 51, 52. Most importantly, it has been found that miR‐10a was regulated in immune cells and involved in autoimmune diseases *via* restricting inflammation. Down‐regulated expression of miR‐10a while increased expression of NOD2 and IL‐12/IL‐23p40 in the inflamed mucosa of patients with IBD was reported recently 37. TNF‐α, IFN‐γ and commensal bacteria inhibited miR‐10a expression in human DC, and miR‐10a was found to distinctly suppress Th1 and Th17 cell responses in IBD. Another study showed that microbiota negatively regulated miR‐10a expression of intestinal epithelial cells and DC, which might contribute to the maintenance of intestinal homeostasis by targeting IL‐12/IL‐23p40 expression 38. Also, miR‐10a is essential for Treg stability and function. It was expressed in Tregs but not in other T cells 39. It has been found that miR‐10a expression was increased by retinoic acid and TGF‐β in Treg cells, which attenuated the phenotypic conversion of Treg into follicular helper T cells *via* targeting the transcriptional repressor Bcl6 and the corepressor NCOR2 40. Notably, miR‐10a‐5p recently was found to be dysregulated in the FLS of rheumatoid arthritis 41. Here we also detected miR‐10a‐5p expression regulation in the synovium of patients with RA, and significant down‐regulation in RA was confirmed compared with OA control. However, the dysregulation mechanism of miR‐10a‐5p as well as its downstream targets and function in FLS remains poorly understood, which became the intention for us to design and perform further experiments.

From previous T cell–FLS co‐culture experiment, TNF‐α from T cells contributes to TLR3 regulation in FLS. Likewise, arthritogenic T cell‐derived inflammatory factors should also be the primary cause to affect miR‐10a‐5p expression. TNF‐α and IL‐1β considered as candidates were used to stimulate human synoviocyte cell line, to mimic the local inflammatory changes in RA. It has been reported that the stimulation of TNF‐α and IL‐1β could induce the expression of miR‐146a/b and miR‐155 in RA synovial fibroblasts remarkably. Thus, providing the evidence that miRNAs not only contribute to various aspects of RA pathogenesis, but also the expression of miRNAs may be changed by the inflammatory milieu of resident cells in RA joints 20, 53. It is clear that IL‐1β and to some extent TNF‐α could reduce expression of miR‐10a‐5p. But IL‐1β seemed to be more effective for the down‐regulation of miR‐10a‐5p and up‐regulation of its potential target gene TBX5 with time‐ and dose‐dependent manners, whereas TNF‐α showed to reduce TBX5 expression under the unknown mechanism. Thus, we considered that IL‐1β should be the primary cause which might directly regulate miR‐10a‐5p expression in synoviocytes.

Besides the regulation mechanism of miRNA, we next focused on its downstream function in FLS using miRNA mimic and inhibitor. Recently, the intensive research work in the field of miRNAs helps us greatly to understand their role in the regulation of gene expression and their pathological role in various diseases. Abnormal expression of miRNAs has been recently reported in patients with rheumatoid arthritis 54. An elevated expression level of miR‐155 and miR‐146 was found in synovial tissues and synovial fibroblasts isolated from patients with RA 20, 53. The enforced expression of miR‐155 in synovial fibroblasts reduced the expression of MMP‐3 and weakened the induction of cytokines 53. The presence of an elevated level of MMPs is considered as the markers of joint damage and progression of RA 55, 56. It has also been reported that the enforced expression of miR‐19a/b in RA FLS after transfection with its mimic decreased the expression of IL‐6 and MMP‐3, presenting miR‐19a/b as a therapeutic agent for patients with RA to protect from joint inflammation and destruction 11. Our results showed that the overexpression of miR‐10a‐5p in SW982 cells led to the down‐regulation of MMP13, TLR3 and proinflammatory cytokines and chemokines including IL‐6, TNF‐α, IL‐1β and IL‐8 whereas the inhibition of miR‐10a‐5p rescued the expression of aforementioned proinflammatory phenotypes of RA. In the initiation and progression of RA, IL‐6, IL‐1β and TNF‐α play a very critical role and act as strong pathogenic regulators 57, 58. IL‐8 is an important chemokine which acts as a chemoattractant for neutrophils and is also a potent angiogenic factor 59. TLRs play a crucial role in the progressions of RA in response to PAMP and DAMP located in the joints of patients with RA, and miRNAs have appeared as a new class for the regulation of TLRs. It has been reported that TLR3 became up‐regulated due to decreased expression level of miR‐26a in the spleen of PIA rats 60. Locally, we found that TNF‐α induced TLR3 expression in FLS *via* activation of p38 MAPK and NF‐κB pathway 36. Our present findings on miR‐10a‐5p negatively regulating TLR3 in FLS could be a supplementary mechanism. Subsequently, FLS upon stimulation with TLR3 produce Type I interferons which possess inflammatory functions 61. Overall, it can be concluded that the silencing of miR‐10a‐5p plays a paramount role in arthritis by regulating and promoting proinflammatory cytokines, TLR3 and MMP13 as well.

Mechanistically, how miR‐10a‐5p regulated FLS function needed to be further addressed. We searched the target genes of miR‐10a‐5p using the bioinformatics tools, and TBX5 appeared as a potential target gene, which had also been confirmed to be up‐regulated in both RA synovium and IL‐1β stimulated synoviocytes in our preliminary experiments. TBX5 is a well‐known transcription factor and a member of T‐box transcription factor family; in which each candidate possesses a common T‐box DNA‐binding domain in their sequences. TBX5 plays an important role in tissue development and Cancer. Genetic mutations in the TBX5 sequence have been reported with the Holt–Oram syndrome, which involves dysfunctions in the heart and limbs 62. Recently, TBX5 was illustrated as a novel tumour suppressor and assessed for its potential use as a biomarker for colon cancer 63. In particular, TBX5 is an important transcription factor which regulates the synovial fibroblast and induces the secretion of inflammatory cytokines in RA 64. TBX5 was less methylated in RA synovium and RA FLS than in OA samples. Demethylation of the TBX5 promoter in RA FLS and RA synovium showed higher TBX5 expression than in OA FLS and OA synovium 64. These results convinced that TBX5 could be involved in the development of RA, but its regulation remains unclear.

We constructed luciferase reporter plasmid and verified that miR‐10a‐5p could directly target TBX5. What is more, transfection of miRNA mimic or inhibitor could reduce or increase TBX5 expression in FLS, respectively. Further, we were also interested in investigating the possible role of TBX5 in synoviocytes, and especially whether TBX5 mediated miR‐10a‐5p induced functional changes. Overexpression or Knocking down of TBX5 in IL‐1β‐stimulated synoviocytes successfully induced or reduced the expression of TLR3, MMP13 and proinflammatory cytokines which had been proven to be regulated by miR‐10a‐5p.

Taking together, we concluded that down‐regulation of miR‐10‐5p in synoviocytes of RA promotes the expression of TBX5, which in turn increases the expression of inflammatory effectors and participates in the development of an inflammatory process of RA.

## Conflict of interest

None of the authors have any potential financial conflict of interest related to this manuscript.
